# Injury registration for primary prevention in a provincial Russian region: setting up a new trauma registry

**DOI:** 10.1186/s13049-019-0627-1

**Published:** 2019-04-17

**Authors:** Tatiana N Unguryanu, Andrej M Grjibovski, Tordis A Trovik, Børge Ytterstad, Alexander V Kudryavtsev

**Affiliations:** 1 Department of Community Medicine, UiT - The Arctic University of Norway, Hansine Hansens veg 18, 9019 Tromsø, Norway; 2 Arkhangelsk International School of Public Health, Northern State Medical University, Troitsky Ave., 51, Arkhangelsk, 163000 Russia; 3 North-Eastern Federal University, Belinsky str., 58, Yakutsk, 677027 Russia; 4 Al-Farabi Kazakh National University, Al-Farabi Ave., 71, Almaty, 050040 Kazakhstan

**Keywords:** Injury registry, Shenkursk, Completeness, Reliability

## Abstract

**Background:**

The Shenkursk Injury Registry (SHIR) was established in the Shenkursk District, Northwestern Russia in 2015 for the purposes of primary prevention. The SHIR covers all injuries (ICD-10 diagnoses from S00 to T78) for which medical aid is given at the Shenkursk central district hospital and includes data about injury circumstances. We used the SHIR data to assess the quality of the SHIR as an evidence basis and for the local preventive applications.

**Methods:**

Completeness, representativeness, and reliability of the SHIR data were assessed using a sample of 1696 injuries which have occurred in July 2015–June 2016. Chi-square tests were used to assess differences between the registered and missed cases in the registry and Cohen’s kappa were applied to assess the agreement between independent data entries.

**Results:**

The completeness of the SHIR with respect to the coverage of cases treated at the Shenkursk central district hospital was 86%. There were no differences between the registered and the missed injuries by sex, ICD-10 codes, weekday of admission, but there were differences in their distribution by attending physicians. Also, higher proportions of child injuries and injuries in the summer time were among the missed cases. Signs of lower injury severity (different distribution by ICD-10 codes and lower proportion of traffic injuries) were observed among injuries in rural areas which were not covered by the registry because of treatment at rural primary health care units without referrals to the central hospital. Two independent data entries from standard paper injury registration forms showed a 79–99% agreement, depending on the variable considered.

**Conclusion:**

With consideration of possible insubstantial overestimates of the average injury severity, the SHIR data can be considered sufficiently complete, reliable, and representative of the injury situation in the Shenkursk District. Therefore, SHIR is an adequate evidentiary basis for planning local injury prevention.

## Background

Injuries are the third leading cause of death in the European region, after diseases of the circulatory system and neoplasms [1, 2]. The same applies to the Russian Federation, but its injury-related mortality (126.8 per 100,000) is far higher than that in any other European country [3]. Indeed, the age-standardized mortality rate from external causes in Russia is 1.4 times higher than that in Kazakhstan (88.5 per 100,000), Russia’s neighbor to the South, and more than three times higher than that in Norway (37.3 per 100,000) to the North-West [3].

International evidence shows that good-quality injury data are a prerequisite for effective prevention [4–6]. Therefore, collection of injury data through surveillance systems or injury registries should be the first step in the planning of preventive activities [7, 8]. Injury registries are databases that document injuries in specified areas according to defined inclusion criteria and variables. In addition to their use in prevention, injury registries can be used for policy development, improvement of the quality of injury care, clinical and epidemiological research [5, 9].

In Russia, injuries, poisonings, and other consequences of external causes are registered by the receiving hospitals. For an injury, the hospital information systems record the code according to the International Statistical Classification of Diseases, 10th Revision (ICD-10) [10], patient’s age, sex, time and place of treatment as well as data on treatment provided. Each year, all hospitals pass on their collected information to the Federal State Statistics Service, which creates summaries and reports of the data [11–13]. This system allows a comprehensive descriptive overview of medically-treated injuries, but it has limited use for primary prevention because it lacks information about when, where, and how injuries occur.

A number of countries have gone a step further and created injury registration systems which aim to prevent injuries. Such injury registries and databases have played an important role in lowering injury incidence, injury-related mortality and disabilities [4, 14, 15]. For example, the Harstad Injury Registry in Norway has been systematically collecting injury data for more than 30 years, including detailed records of injury circumstances, mechanisms, and involved factors [6, 16, 17]. The registry data serves an evidence basis for the Harstad Safe Community Program and has been metaphorically called “the locomotive that keeps the injury prevention train on its track” [6, 15, 17]. Based on the experience of the Harstad Injury Registry and with similar purposes, the population-based Shenkursk Injury Registry (SHIR) was established in the Shenkursk District, Northwestern Russia in 2015.

The aims of this study were to assess the quality of the SHIR data for use as an evidence basis and for local preventive applications.

## Methods

### Study site

**Fig. 1:**
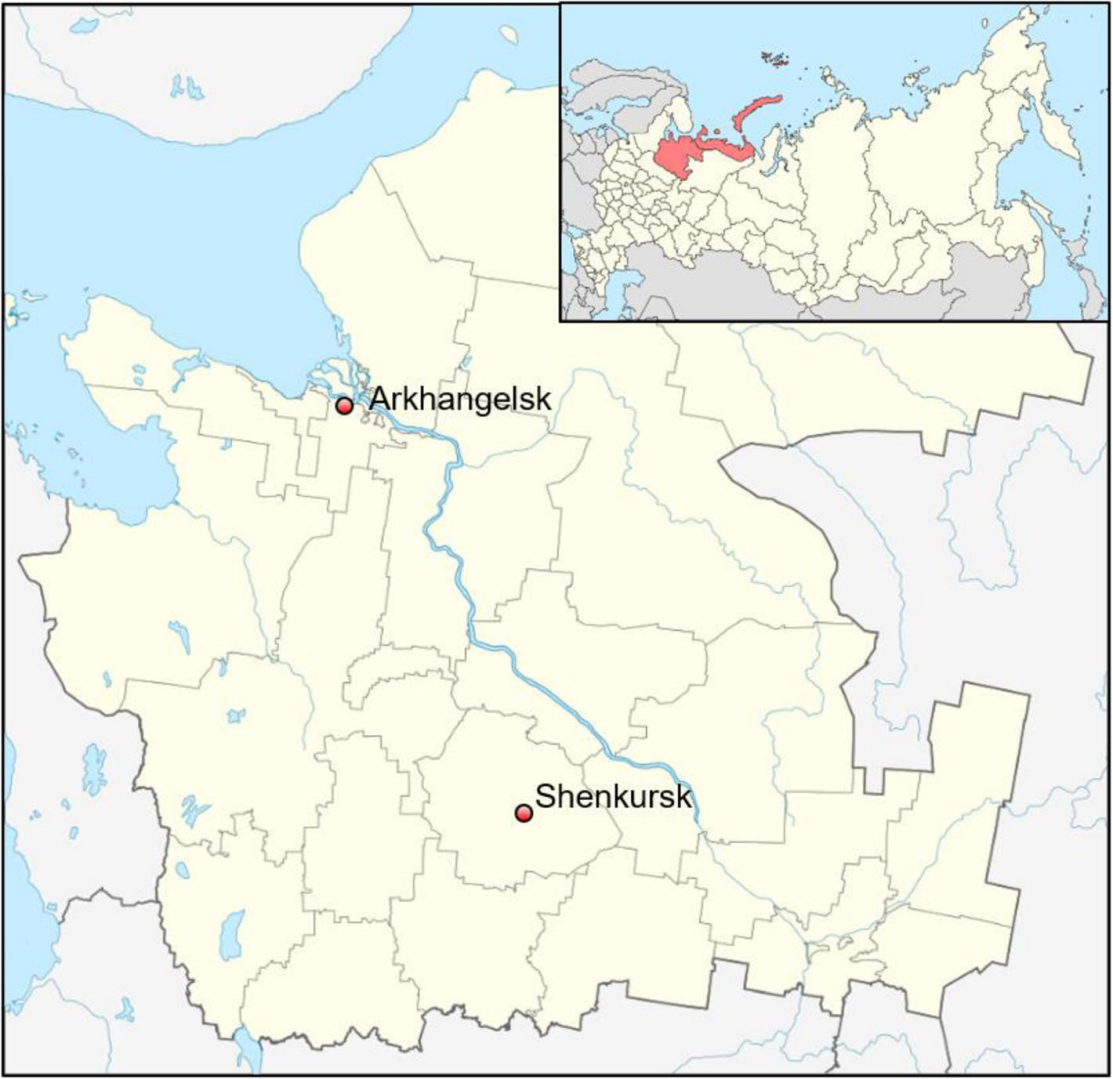
Location of the town of Shenkursk in the Arkhangelsk Region, Russia, 2018 (Source: https://en.wikipedia.org/wiki/Shenkursk)

The Shenkursk District of the Arkhangelsk Region, Northwestern Russia (Fig. 1) had a population of 13,530 on 1 January 2015. The town of Shenkursk (*n* = 5073) is the administrative center of the district and its only urban settlement [18]. It is situated 380 km of Arkhangelsk City, near the M8 Russian Federal highway that links Arkhangelsk and Moscow.

Health care in the district comes from two sources: the central district hospital and rural primary care units. The central district hospital is located in the town of Shenkursk and has in-patient departments (62 beds) as well as adult and pediatric outpatient units. The rural primary care units include two out-patient departments and 23 feldsher-midwife stations. Records of treatment at all of these locations are held at the hospital information system of the Shenkursk central district hospital, according to Russian national healthcare standards.

### The Shenkursk injury registry

The SHIR was created using the translated manual for the Harstad Injury Registry [6, 15, 17]. The standard injury registration form (IRF) of the SHIR is developed on the basis of the form used in Harstad Injury Registry, and the registration instructions and coding lists for free-text variables are the translations of those used in Harstad [19]. Before launching the SHIR, pilot tests were done on the IRF, data collection logistics, the data management system; and two nurses were trained as injury registrars and were tested on quality of data entry [19].

The SHIR is intended to cover all injuries (ICD-10 diagnoses from S00 to T78) for which medical aid is given at the Shenkursk central district hospital. The data are initially collected using paper-based IRFs - two-page sheets with sections for recording patients’ socio-demographic characteristics (sex, date of birth, address of residence, place of work or study), information about time and place of the injury, alcohol consumption in the 24 h before injury, use of protective equipment, and optional sections for descriptions of road traffic and sport injuries. The IRF also has a free-text field for recording a verbal description of how the injury occurred. This field is supplemented by three supportive questions to facilitate such descriptions: “What were you doing?”, “What went wrong?”, “How were you injured?”. This is consistent with the concepts of pre-crash, crash, and post-crash described by Haddon [20] as well as with the Nordic System [21], facilitating analysis of the injury panorama and targeting local injury prevention. The concluding part of the form has several fields to be completed by a physician: diagnosis, ICD-10 code, injury severity according to the Abbreviated Injury Scale (AIS) [22], generalized cause of injury (accident, violence, self-inflicted harm), hospitalization (yes/no), and the name of attending physician.

Physicians who provide treatment for injuries at the Shenkursk central district hospital or in its ambulance cars are instructed to offer the IRF to each treated patient at their first outpatient or ambulance visit, or within few days after hospitalization. Patients complete the IRFs, often with the assistance of accompanying relatives, a nurse, or a physician. If a patient does not complete the IRF due to a severe condition or other reasons, injury registrars (the two trained nurses) complete the form retrospectively, using data from routine medical records (ambulance journal, outpatient medical card, case history) as well as information obtained from the attending physician.

Once the IRF is completed, its data is manually entered into the SHIR database (based on Epi Info 7) [23] by the two injury registrars. Following the translated Harstad manuals, at this stage of processing the IRFs a series of coding lists are used to transform the free-text descriptions of the injury situations into several categorical variables: type of injury site (place of injury), mechanism of preceding activity, mechanism of accident, mechanism of injury, and three variables to record factors involved at the three phases of an injury event, accordingly. The registrars have detailed instructions on how to enter raw data from paper-based injury registration forms into Epi Info and are calibrated and on how to transform free-text descriptions of injury situations into the categorical variables.

### Data analysis

One year of the SHIR data (1 July 2015–30 June 2016, 1696 registered injuries) was used for assessments of the data quality, i.e. data completeness, representativeness, and reliability. The completeness in terms of coverage of injuries treated at the Shenkursk central district hospital was assessed by linkage of the SHIR records to those of the hospital information system, assuming that the latter included all injuries. Record linkage of the SHIR and the hospital system was performed using names (recorded in the hospital system; available for the SHIR records on corresponding paper IRFs), dates of birth, and dates of injuries.

The representativeness of the SHIR data for the Shenkursk District was assessed by comparing registered injuries to those treated at the Shenkursk central district hospital but not registered in the SHIR. As the second step, registered injuries that occurred in rural areas (i.e. rural injuries) and were treated at the Shenkursk central district hospital were compared to rural injuries that were treated at primary care units, and thus have fallen outside the SHIR’s coverage. These comparisons were performed based on variables that are present in the hospital system: age, sex, ICD-10 code, and time variables for initial hospital visit (month of year, day of week). Chi-square tests were used to assess differences between the compared groups.

Data reliability in the SHIR was assessed as agreement between the routine data entry from IRFs performed by the two injury registrars; and a second entry that was independently performed by the first author. This second entry included independent coding of original text descriptions of injury circumstances, following the same coding lists and procedures. Variables used for reliability assessments were sex, age, ICD-10 code, date of injury, mechanism of preceding activity, accident mechanism, injury mechanism, factors involved in each of the mechanisms, alcohol consumption in the 24 h before injury, generalized injury cause, AIS [22], and hospitalization. Cohen’s kappa was used to assess the agreement.

All statistical analyses were performed using SPSS, version 24 (SPSS Inc., Chicago, IL, USA).

## Results

**Fig. 2:**
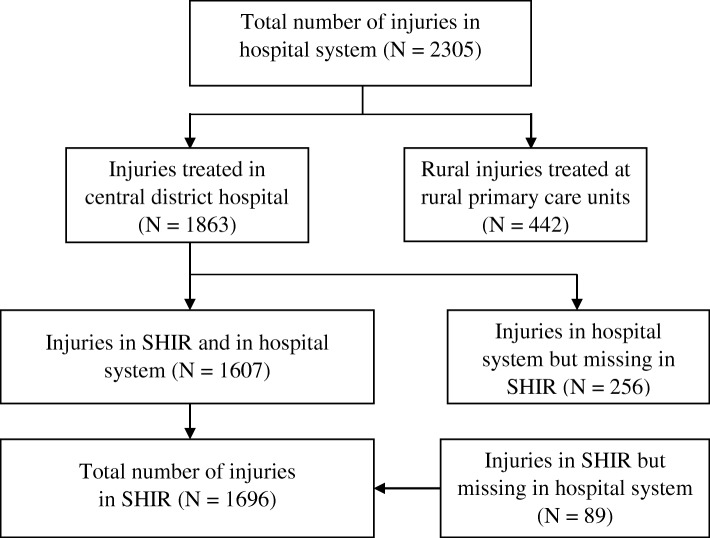
Distribution of injuries in the hospital system of the Shenkursk central district hospital and in the SHIR, July 2015–June 2016

According to the hospital information system, there were 2305 injuries in the Shenkursk District between July 2015 and June 2016 (Fig. 2). Among them, 1863 injuries were treated at the Shenkursk central district hospital and 442 at rural primary care units (i.e. outside the SHIR’s coverage). Of the 1863 injuries treated in the central district hospital, the SHIR included 1607 (86.3%) and missed 256 cases, reflecting the data completeness. Moreover, there were 89 injuries included in the SHIR and were missing in the hospital system. That gave a total of 1696 injuries registered in the SHIR in July 2015–June 2016.

There were no differences between the 1607 registered injuries and the 256 missed injuries by sex, ICD-10 diagnostic (S00-T78) and external cause (V01-Y98) codes, or weekday of admission (Table 1). The proportions of injuries occurring in children and in the summer time were higher among the missed injuries compared to the registered ones. Also, missing and registered injuries had different distribution by attending physicians.

**Table 1:** Comparison of injuries registered and missed in the Shenkursk Injury Registry, July 2015–June 2016 (*n* = 1863^a^)

Variables	Registered injuries (*N* = 1607)		Missed injuries (*N* = 256)		p
	N	%	N	%	
Sex					
Male	942	58.6	153	59.8	0.729
Female	665	41.4	103	40.2	
Age					
0–17 years	382	23.8	87	34.0	< 0.001
18+ years	1225	76.2	169	66.0	
Injury localization, ICD-10					
S00–09: Head	282	17.5	53	20.7	0.635
S20–29: Thorax	107	6.7	13	5.1	
S40–49: Shoulder and upper arm	109	6.8	16	6.3	
S50–59: Elbow and forearm	130	8.1	21	8.2	
S60–69: Wrist and hand	248	15.4	34	13.3	
S80–89: Knee and lower leg	152	9.5	30	11.7	
S90–99: Ankle and foot	207	12.9	27	10.5	
T15–19: Foreign body entering through natural orifice	153	9.5	19	7.4	
Others codes^b^	435	27.0	72	28.1	
External causes, ICD-10					
W00-W19: Slipping, tripping, stumbling and falls	592	36.8	87	34.0	0.612
W20-W49: Exposure to inanimate mechanical forces	518	32.2	94	36.7	
W50-W64: Exposure to animate mechanical forces	167	10.4	26	10.2	
Others codes^b^	160	10.0	25	9.7	
Missing data	170	10.6	24	9.4	
Season of year					
Winter	368	22.9	54	21.1	0.001
Spring	448	27.9	33	12.9	
Summer	430	26.8	117	45.7	
Autumn	357	22.5	52	20.3	
Weekday					
Monday	289	18.0	42	16.4	0.600
Tuesday	276	17.2	34	13.3	
Wednesday	266	16.6	41	16.0	
Thursday	237	14.7	34	13.3	
Friday	281	17.5	53	20.7	
Saturday	148	9.2	24	9.4	
Sunday	106	6.6	18	7.0	
Attending physicians					
Dr.A	10	0.6	36	14.1	< 0.001
Dr.B	92	5.7	9	3.5	
Dr.C	20	1.2	25	9.8	
Dr.D	379	23.6	55	21.5	
Dr.E	159	9.9	9	3.5	
Dr.F	111	6.9	7	2.7	
Dr.G	128	8.0	8	3.1	
Others^b^	708	44.1	107	41.8	

^a^All analyzed injuries were treated in the Shenkursk central district hospital, as shown in the hospital system for accounting of medical services; ^b^Combines categories accounting for < 5% of observations

*ICD* International Classification of Diseases

Of the 1696 injuries registered in the SHIR in July 2015–June 2016, 610 were rural injuries which received treatment at the central district hospital. As indicated above, the hospital system contained data for another 442 rural injuries treated at rural primary care units which are not covered by the SHIR. Rural injuries treated at rural primary care units did not differ from those treated at the central district hospital and registered in the SHIR by sex and season of the year, but a higher proportion of rural injuries were represented by children and by injuries to the wrist and hand. A lower proportion of rural injuries treated in primary care were represented by injuries to the shoulder and upper arm (Table 2). Besides, the rural injuries treated in primary care were less commonly caused by transport accidents, and more commonly resulted from exposure to animate mechanical forces.

**Table 2:** Comparison of rural injuries treated at the Shenkursk central district hospital and rural injuries treated at rural primary care units of Shenkursk district, July 2015–June 2016 (*N* = 1052)

	Rural injuries treated at central district hospital (*N* = 610)		Rural injuries treated in primary care (*N* = 442)		p
	n	%	N	%	
Sex					
Male	359	58.9	239	53.6	0.088
Female	251	41.1	207	46.4	
Age					
0–17 years	125	20.5	152	34.1	< 0.001
18+ years	485	79.5	294	65.9	
Injury localization, ICD-10					
S00–09: Head	77	12.6	66	14.8	< 0.001
S20–29: Thorax	54	8.9	28	6.3	
S30–39: Abdomen, lower back, lumbar spine, pelvis and external genitals	30	4.9	29	6.5	
S40–49: Shoulder and upper arm	64	10.5	13	2.9	
S50–59: Elbow and forearm	56	9.2	21	4.7	
S60–69: Wrist and hand	83	13.6	89	20.0	
S80–89: Knee and lower leg	72	11.8	47	10.5	
S90–99: Ankle and foot	77	12.6	68	15.2	
T15–19: Foreign body entering through natural orifice	28	4.6	26	5.8	
T20–32: Thermal and chemical burns	33	5.4	19	4.3	
Others codes^a^	36	5.9	40	9.0	
ICD-10 external cause codes					
V01-V99: Transport accidents	37	6.1	11	2.5	< 0.001
W00-W19: Slipping, tripping, stumbling and falls	237	38.9	173	38.8	
W20-W49: Exposure to inanimate mechanical forces	171	28.0	141	31.6	
W50-W64: Exposure to animate mechanical forces	46	7.5	58	13.0	
Others codes^a^	36	6.0	44	10.0	
Missing data	83	13.6	19	4.3	
Season of year					
Winter	140	23.0	110	24.9	0.812
Spring	163	26.7	114	25.8	
Summer	193	31.6	131	29.6	
Autumn	114	18.7	87	19.7	

^a^Combines categories accounting for < 5% of observations

*ICD* International Classification of Diseases

The agreement between two independent data entries for date of birth, date of injury, date of IRF completion, sex, ICD-10 code, alcohol consumption in the 24 h before injury, generalized cause of an injury, AIS, and hospitalization was at the level of 98–99% (Table 3). The agreement in variables that result from coding of the free-text descriptions of injury situations and reflect mechanisms of preceding activities, accidents, and injuries ranged between 91 and 95%. The agreement in variables resulting from coding of factors involved at three phases of injury events ranged from 79 to 88%.

**Table 3:** Agreement between the two independent data entries for the Shenkursk Injury Registry (*N* = 1696)

Variables	Agreement		Cohen’s Kappa
	n	%	
Date of birth	1687	99.5	–
Date of injury	1682	99.2	–
Date of form filling	1673	98.6	–
Sex	1965	99.8	0.99
ICD-10 diagnostic сode	1680	99.1	0.99
Alcohol consumption	1678	98.9	0.98
Generalized cause of injury	1668	98.3	0.94
Injury severity (AIS)	1685	99.4	0.99
Hospitalization	1690	99.6	0.98
Mechanism of preceding activity	1536	90.6	0.89
Accident mechanism	1605	94.6	0.94
Injury mechanism	1589	93.7	0.93
Factor 1 (preceding activity)	1343	79.2	0.77
Factor 2 (accident)	1487	87.7	0.87
Factor 3 (injury)	1415	83.4	0.83

*ICD* International Classification of Diseases, *AIS* Abbreviated Injury Scale.

## Discussion

Injury registries exist in the USA, Australia, Canada, Norway, Germany, and the UK [17, 24], but to our knowledge, the SHIR is the first injury registry in Russia.

Most national or regional injury registries are limited to trauma center hospitals and include only severe injuries (for example, “major trauma” or persons hospitalized for more than 24 h, etc.) [25]. On the contrary, the SHIR is population-based as it covers all injuries treated at the only hospital in Shenkursk District. Moreover, the SHIR collects detailed information on injury circumstances. These two features target the SHIR’s representativeness of the total number of injuries in the coverage area and form an informative evidence basis for primary prevention, according to recommendations for injury monitoring and prevention [4, 26, 27].

### Methodological considerations

This study has shown that the completeness of the SHIR with respect to the coverage of cases treated at the central district hospital was 86%, which is lower than that reported from several other registries. For example, Thailand has a provincial injury surveillance system that covers injuries from five large hospitals and has a reported completeness of 98.8% [28], and the trauma registry in Peru includes a reported 99% injuries admitted to the main referral hospital [29]. However, these studies did not specify how completeness was calculated. On the other hand, a similar completeness (90%) was reported in a study of hip fractures in the Harstad Injury Registry in Norway [16].

The approach we used to assess completeness of the SHIR assumed that the hospital system covers all injuries treated in the central district hospital. However, we identified a number of injuries in the SHIR that were treated in the hospital but were missing in the hospital system for unknown reasons. That may indicate that our estimates may be slightly underestimated.

The imperfect completeness of the SHIR may be primarily explained by the difference between missed and registered injuries in their distribution by attending physicians, which reflects unequal efficiency of physicians to collect IRFs from their patients. Practically, injuries with uncompleted IRFs are largely detected by injury registrars through regular checks of the registry against the hospital system. In such cases, the registrars complete the IRFs retrospectively, using available medical documentation and information obtained from physicians. In order to assess the proportion of IRFs competed by registrars, in 2017 the SHIR was added by a variable specifying who completed the IRF. In 2017, 59% of IRFs were completed by patients or accompanying relatives, and 41% were filled in by registrars. This reflects the registrars’ substantial efforts to achieve the highest possible completeness, although the results are still imperfect. Based on that and in order to improve the completeness of the SHIR, the administration of the hospital was advised to enhance physicians’ motivation to ask their patients to complete IRFs.

According to the information in the hospital information system, there were no substantial differences between registered and missed injuries in the SHIR by sex, weekday of admission, diagnostic and external cause categories, but missed injuries showed a higher proportions of children (34% vs. 24%) and higher proportion of injuries occurring in summer time (46% vs. 27%). The increased probability of failure to register injuries in summer may be explained by the vacation period, when a part of the staff is absent and those remaining must deal with a higher volume of prioritized medical tasks. The higher proportion of missed injuries among children may also be due to higher underregistration in summer time, when children have holidays and are more likely to get injured.

As the proportion of missed injuries in the SHIR was 14% and the missed cases showed only minor differences from those registered, the imperfect completeness of the registry should not substantially affect its representativeness for total injuries treated at the central district hospital. However, there are some underestimates of injury risks among children and in summer time, which should be taken into account when using the data for preventive purposes.

The population covered by the SHIR is rather small (*n* = 13,530). This implies that sampling of specific injuries for research purposes may take a long time, or confidence intervals may be broad. However, the primary aim of the SHIR is to identify circumstances, mechanisms and involved factors of the most frequent injuries in the Shenkursk district for planning targeted district-level prevention. Studies of these injuries are expected to have good levels of statistical power. And, the longer the registry is run, the more cases are generated.

A limitation of the SHIR is that it only covers injuries that received medical aid at the Shenkursk central district hospital while about 40% of injuries occurring on adjacent rural areas are treated at primary care units and thus are not recorded in the SHIR. Comparisons of rural injuries treated at the central district hospital to those treated at rural primary care units reflect the real-life situation: more severe cases (e.g. those resulting from traffic accidents) are more commonly referred to the central district hospital, while minor injuries are more often treated at primary care units. The described differences between the two groups were significant but not decisively substantial, so the SHIR data can be considered fairly representative of all injuries in the district. Although, a notation of some overestimations in overall injury severity has to be made.

The difference in coverage of urban and rural injuries by the SHIR also creates difficulty in estimating the incidence. On one hand, not all cases appear in a numerator if we calculate the incidence for the whole district, thus leading to an underestimate. An alternative way can be to limit the numerator to cases in the town, and use the town’s population in denominator. But this reduces statistical power and does not represent the situation in the entire district with large rural component. This data deficiency can be compensated by calculating the incidence using the information of hospital system, which covers all rural and urban cases. This would lead to more precise incidence estimates by sex, age or ICD-10, while the registry provides valuable information about typical injury circumstances.

Another limitation is the non-inclusion of prehospital fatal injuries. The reason for that is that such fatalities are referred to a forensic department outside the hospital. Thus, the SHIR cannot be used to estimate the burden and describe the characteristics of fatal injuries. This weakness has to be considered when using the SHIR data for preventive applications, but its elimination would unlikely affect the registry-derived priorities for preventive interventions as fatal injuries constituted, for example, only 2.1% of the total injuries in the Shenkursk District in the study period from July 2015 to June 2016.

In the present study, we expressed data reliability as percentage of agreement between two independent raters. The data value was considered reliable if it was the same for the two raters. According to the classification by Landis and Koch, the agreement was “almost perfect” for variables resulting from simple data entry from IRFs into the SHIR (e.g. date of birth, sex, ICD-10 code) [30]. For variables resulting from free-text descriptions through coding procedures (e.g. mechanism of accident, factor involved in mechanism of accident), the agreement levels ranged from “substantial” to “almost perfect” (lowest Cohen’s k = 0.77). More frequent disagreements in these variables may be explained by possible variations in the subjective understanding of free-text descriptions and the corresponding codes assigned. Such reliability deficiencies are unavoidable, and their relatively small volumes show that the SHIR data is reasonably reliable.

The presented data reliability assessments referred to the stage when the data is transferred manually from paper-based IRFs into the electronic registry, but the reliability of IRF data can scarcely be assessed. Potential deficiencies were addressed during staff training and in the design of the form. For example, the correct indication of injury severity according to the AIS in the medical part of the form requires a physician to be familiar with this scale. To prevent possible errors due to lacking knowledge of the AIS, the IRF itself contains a detailed description of the AIS, in order to facilitate proper ranking by physicians.

## Conclusion

This study has demonstrated that the SHIR can be considered sufficiently complete, accurate, and representative of the injury situation in the district. With a notation of a possible insubstantial overestimate of the average injury severity in the district, it forms a suitable evidence basis for local preventive activities and can be used for injury research.

## Abbreviations

AIS: Abbreviated Injury Scale
ICD: International Statistical Classification of Diseases and Related Health Problems
IRF: Injury registration form
SHIR: Shenkursk Injury Registry
WHO: World Health Organization
